# Genomic risk prediction of aromatase inhibitor‐related arthralgia in patients with breast cancer using a novel machine‐learning algorithm

**DOI:** 10.1002/cam4.1256

**Published:** 2017-11-23

**Authors:** Raquel E. Reinbolt, Stephen Sonis, Cynthia D. Timmers, Juan Luis Fernández‐Martínez, Ana Cernea, Enrique J. de Andrés‐Galiana, Sepehr Hashemi, Karin Miller, Robert Pilarski, Maryam B. Lustberg

**Affiliations:** ^1^ The Ohio State University Comprehensive Cancer Center Columbus Ohio; ^2^ Primary Endpoint Solutions Watertown Massachusetts; ^3^ Brigham and Women's Hospital and the Dana‐Farber Cancer Institute Boston Massachusetts; ^4^ University of Oviedo Oviedo Spain; ^5^ Harvard School of Dental Medicine Boston Massachusetts

**Keywords:** Aromatase, arthralgia, breast cancer, estrogen, joint pain, SNP

## Abstract

Many breast cancer (BC) patients treated with aromatase inhibitors (AIs) develop aromatase inhibitor‐related arthralgia (AIA). Candidate gene studies to identify AIA risk are limited in scope. We evaluated the potential of a novel analytic algorithm (NAA) to predict AIA using germline single nucleotide polymorphisms (SNP) data obtained before treatment initiation**.** Systematic chart review of 700 AI‐treated patients with stage I‐III BC identified asymptomatic patients (*n* = 39) and those with clinically significant AIA resulting in AI termination or therapy switch (*n* = 123). Germline DNA was obtained and SNP genotyping performed using the Affymetrix UK BioBank Axiom Array to yield 695,277 SNPs. SNP clusters that most closely defined AIA risk were discovered using an NAA that sequentially combined statistical filtering and a machine‐learning algorithm. NCBI PhenGenI and Ensemble databases defined gene attribution of the most discriminating SNPs. Phenotype, pathway, and ontologic analyses assessed functional and mechanistic validity. Demographics were similar in cases and controls. A cluster of 70 SNPs, correlating to 57 genes, was identified. This SNP group predicted AIA occurrence with a maximum accuracy of 75.93%. Strong associations with arthralgia, breast cancer, and estrogen phenotypes were seen in 19/57 genes (33%) and were functionally consistent. Using a NAA, we identified a 70 SNP cluster that predicted AIA risk with fair accuracy. Phenotype, functional, and pathway analysis of attributed genes was consistent with clinical phenotypes. This study is the first to link a specific SNP/gene cluster to AIA risk independent of candidate gene bias.

## Introduction

Aromatase inhibitors (AIs) are critical to the management of women with hormone receptor‐positive breast cancer (BC). Their use has evolved to include premenopausal women with high‐risk BC in combination with ovarian suppression 1, 2. Whether administered as monotherapy, sequential therapy, or extended therapy, AIs favorably impact disease free survival 3. However, AIs are also associated with a number of toxicities, of which arthralgia (AIA) is among the most common and significant.

The broad range of reported incidence of AIA (5–47%) may be attributed to a lack of uniformity in diagnostic criteria to define the condition and casual reporting 4. In two studies specifically designed to identify AIA, the incidence of AIA was consistently reported near 50%, and did not vary across different third‐generation AIs 5, 6. Importantly, AIA frequently results in noncompliance with AI regimens or treatment discontinuation entirely, both of which adversely impact clinical outcomes 5, 7.

Several clinical risk factors for AIA have been reported, including time since last menstrual cycle 8, obesity, prior hormone replacement therapy 4 or chemotherapy, particularly taxanes 6. The relationship between low estrogen levels and arthralgias has been well reported 9. Niravath argues that an inflammatory intermediary is a driver of low estrogen and AIA 4. However, a precise predictor of women most at risk for developing AIA is not yet established.

Several studies have assessed a potential genomic link to AIA risk 10, 11, 12. Aside from a common methodological approach relying on prospective candidate gene or single‐nucleotide polymorphism (SNP) selection for study, these investigations’ conclusions have varied. Despite such inconsistencies, we believe an alternative analytical approach may effectively identify germline mutations associated with an increased risk of moderate to severe forms of AIA. Furthermore, we believe gene attribution of these mutations might elucidate the pathophysiology underlying AIAs.

We devised a case–control study of women with early stage breast cancer with and without significant AIA. We favored methodology recognizing that risk amplification was more likely when groups of synergistically expressed SNPs occurred (rather than a single SNP) – a departure from the ‘magic bullet’ approach. Consequently, we adapted a novel analytical method, previously proven to be effective in a smaller number of patients, to identify an optimum cluster of SNPs that when expressed together, differentiate those patients at risk for AIA 13.

## Patients and Methods

After Institutional Review Board permission was obtained, a systematic chart review of women enrolled in The Columbus Breast Cancer Tissue Bank was completed to identify patients receiving first‐line adjuvant treatment with third‐generation AIs (anastrazole, exemestane, letrozole) for at least 1 month to treat stage I‐III estrogen receptor‐positive BC between 2003 and 2012. Clinical efficacy of endocrine therapy was not captured in this investigation. Concurrent gonadotropin‐releasing (GnRH) agonist therapy, radiation therapy, prior tamoxifen use, and/or chemotherapy were allowed. Patients with metastatic disease or active autoimmune or inflammatory joint disease were excluded. Patients were divided into two groups: those with clinically significant AIA (defined as grade 2 or above by NCI‐CTCv.4 criteria) and/or requiring modification or termination of AI therapy, and those without any reported clinical signs or symptoms of AIA.

Germline DNA was extracted from mononuclear cells at the Human Cancer Genetics Sample Bank, The Ohio State University, according to previously published protocol 14. DNA was quantitated using PicoGreen and 200 ng of each DNA aliquoted into 96‐well plates. After sample elimination for poor quality DNA, SNP genotyping was performed along with appropriate controls. DNA amplification, fragmentation, and hybridization to Axiom UK Biobank genotyping arrays (Affymetrix, Santa Clara, CA) was completed using Axiom Reagent Kits; hybridization, ligation, washing, staining, and scanning of the arrays was completed on the GeneTitan MC instrument (Affymetrix). Initial plate QC was performed using Affymetrix Genotyping Console Software, and genotype calling done using Affymetrix Power Tools (v1.15.0) with the Axiom GT1 algorithm, which is a modified version of the BRLMM‐P algorithm that adapts generic prior cluster positions to the data using an EM algorithm.

### Analysis

**Figure 1:**
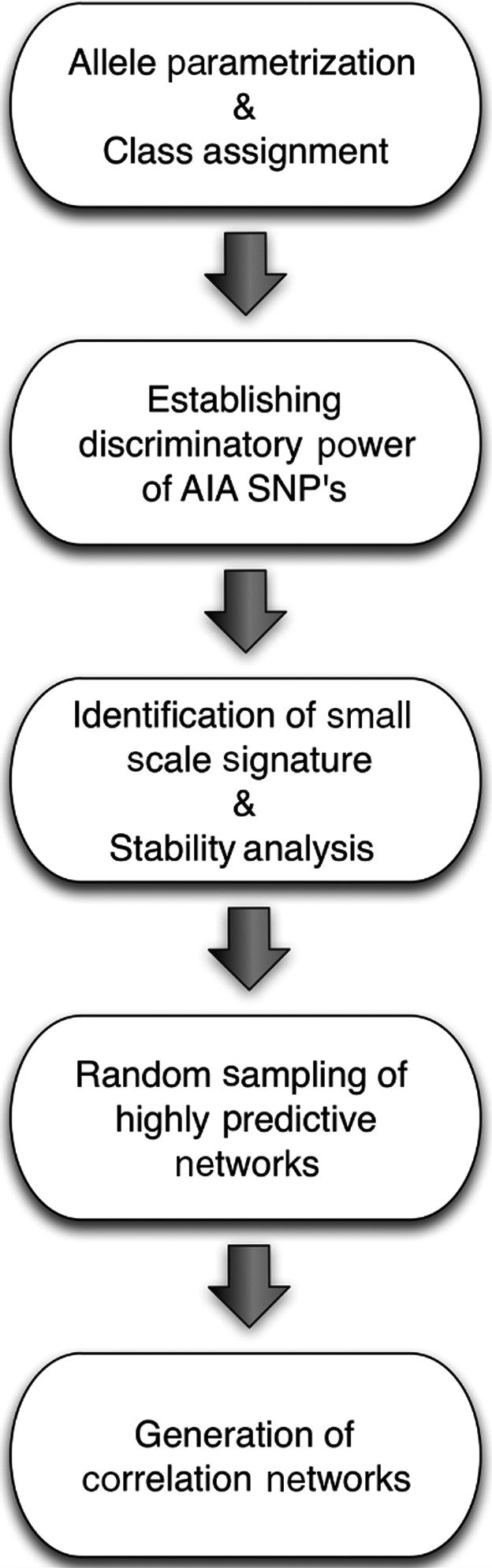
Flowchart of the analytical steps used to identify those SNPs most predictive of AIA risk in the study population.

To establish the parameters by which fold‐change thresholds were maximized to identify distinguishing SNPs, we considered SNPs in 3 groups: those uniquely associated with the AIA‐positive group, the AIA‐negative group, and those predominantly, but not uniquely associated with either group. We then assigned arbitrary numerical identifiers of 1, 2, or 3 to each group for parameterization and found that the maximum fold‐change defining a signal was log2 (3/1) = 1.59. Figure 1 shows the flowchart of the methodology, which is composed of the following steps:

### Analysis of the discriminatory power of the SNPS

To identify the discriminatory power of those SNPs that were differentially noted between the AIA‐positive and ‐negative cohorts, we utilized a machine‐learning algorithm in which we combined fold change and Fisher's ratio. We defined the Fisher's ratio for a SNP *j* in a two‐class classification problem, *c*_1_, *c*_2_ as:

$${\text{FR}}_{j}\left(c_{1},c_{2}\right)=\frac{{\left(\mu_{j^{1}}-\mu_{j^{2}}\right)}^{2}}{\sigma_{j^{1}}^{2}+\sigma_{j^{2}}^{2}}$$

where $\mu_{j^{1}}^{2}$ $\mu_{j^{2}}^{2}$ are measures of the center of the distribution (means) of prognostic variables *j* in classes 1 and 2 (AIA and no AIA), and $\sigma_{j^{1}}^{2}$, $\sigma_{j^{2}}^{2}$ are measures of the dispersion (variance) within these classes. This method is particularly effective for identifying prognostic/predictive variables that separate the classes further apart and are very homogeneous within classes (low intraclass variance).

We considered the 1% tail of the fold change (over and underexpressed SNPs), providing a final set of 436 high discriminatory SNPS with fold change in the interval [−0.31, 0.22] and Fisher's ratio greater than 1.5.

### Finding the small‐scale SNPs signature

Once we identified those SNPs differentiating AIA‐positive and ‐negative patients, we ranked them in decreasing order based on their discriminatory power. Hypothesizing that optimization of predictive risk determination is most accurately the consequence of a collective effect from a cluster of SNPs, we then sought to identify the smallest aggregate of SNPs with the highest prognostic accuracy using an algorithm based on recursive elimination of lower discriminatory SNPs. Our analysis was based on the fact that high discriminatory variables served to span the main features of the classification (AIA), while the variables with lowest discriminatory ratios were of such granularity as to not markedly contribute to being informative as to differential risk. This method determined the minimum amount of high‐frequency details required to optimally discriminate between classes. The predictive accuracy estimation was based on Leave‐One‐Out‐Cross‐Validation (LOOCV) 10, 11 given our goal to estimate how accurately the predictive model (classifier) would perform for future samples with an unknown AIA status.

### Stability analysis of the small‐scale signature

**Figure 2:**
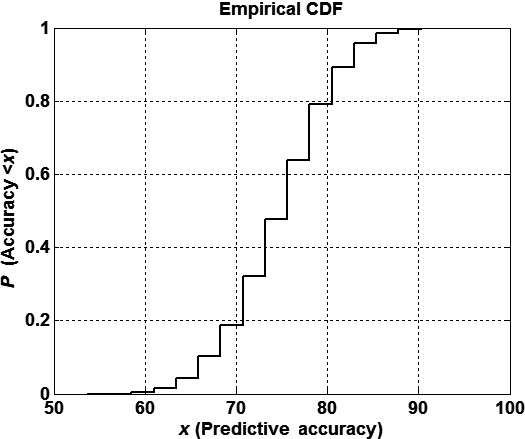
Stability analysis of the small‐scale signature of the 70 SNPs which were identified as being most predictive of AIA risk. The figure shows the cumulative distribution function of the predictive accuracy obtained after 5000 random 75/25 hold out simulations. It can be observed that the median accuracy (percentile 50) is around 75%, being the lower and upper‐quartiles 71% and 78%. The minimum and maximum accuracy achieved was 54% and 100%.

By random 75–25 hold‐out experiments, we next evaluated the stability of the small‐scale signatures’ predictive accuracy (i.e., AIA contributions) found via LOOCV when the number of training samples was decreased. Due to absence of a totally independent clinical data set, the minimum‐scale signature was read in the training dataset for training (75% of the whole set) and applied for blind validation in the validation set (25%). The cumulative distribution function of the small‐scale predictive accuracies found in different hold‐outs was finally presented and accounted for the variability in its predictive accuracy with partial information. An additional statistical analysis was performed to provide the minimum, maximum, and median bounds that could be expected in an independent dataset. Figure 2 shows the cumulative probability function of the predictive accuracy of the small‐scale signature obtained after 5000 random simulations.

### Random sampling of high predictive SNPs equivalent networks

We next used a random sample to find other networks of highly discriminatory, prognostic SNPs. As the prior sampling probability of any individual SNP was considered to be proportional to its Fisher's ratio, we preferentially sampled the most discriminatory SNPs. To look at the impact of SNP synergism in enhancing risk prediction, we developed the most discriminatory networks and analyzed the posterior sampling frequencies of the main prognostic variables involved in each network. The analysis was completed by establishing the correlation network among the most discriminatory SNPs. The network was built using the maximum spanning tree algorithm (an acyclic graph that maximizes the value of the edges) and the Pearson correlation coefficient to identify those SNPS that showed the maximum positive and negative Pearson coefficient.

### Gene attribution and functional analysis

We employed two different databases, NCBI Phenotype‐Genotype Integrator (NCBI PheGenI) 15 and Ensemble release 88 16 using Genome assembly: GRCh38.p9, to gather the genes and their functional consequences associated with the top 70 most discriminatory SNPs (not shown).

To assess the functional validity of the SNP‐associated genes, we performed a comprehensive literature review of each gene associated with the most predictive SNPs, and summarized all relevant phenotypic attributions linking them to the following phenotypes: “arthralgia,” “synovial,” “arthritis,” “rheumatoid,” “joint,” “pain,” “sensitization,” and “nociception.” This was done via an ‘undirected’ review of all publications citing each gene, which was accessed via GeneAnalytics GeneCards’^17^ “Publications” section for each gene of interest 18.

We next determined the presence of any linkage disequilibrium (LD) among the 70 most discriminatory SNPs, as it is plausible that SNPs that individually have marginal influence on arthalgia risk‐associated genes may be inherited in linkage, and together as a group, synergistically increase arthralgia risk. Linkage disequilibrium was assessed by querying the 70 SNPs using Broad Institute's SNP Annotation and Proxy Search (SNAP) tool in the CEU (Utah residents with Northern and Western European ancestry) with *R* ^2^ threshold of 0.8 and distance limit of 500 19, 20.

As a final analysis of functional and phenotypic relevance of the gene set found to be influenced by the 70 SNPs, we applied an undirected assessment using GeneAnalytics software. We reasoned this would provide a relevance validation, but acknowledge the risk of overinterpretation of a small input. This program provides a semiquantitative output of gene cluster relationships relative to disease, pathway, and ontologic relationships. Relationships with “medium” and “high” matched scores for the diseases and pathways were reviewed.

## Results

Systematic chart review of 700 AI‐treated patients with stage I‐III BC identified asymptomatic patients (*n* = 39) and those with clinically significant AIA resulting in AI termination or therapy switch (*n* = 123). There were no significant demographic or disease differences between the AIA‐positive and ‐negative cohorts. Patients were similar in age (controls: 58.6 years, cases: 58.2 year), stage at diagnosis, and estrogen receptor status (Table 1). After sample elimination for poor‐quality DNA, SNP genotyping was performed for 123 AIA positive and 39 AIA negative patients.

**Table 1:** Comparative tumor characteristics of patients in the control (no AIA) and cases (clinically significant AIA) arms

	Controls (*N* = 39) (%)	Cases (*N* = 123) %
Stage I	22 (56)	55 (45)
Stage II	14 (36)	54 (44)
Stage III	3 (8)	14 (11)
ER or PR positive/HER2 negative	33 (85)	104 (85)
ER or PR positive/HER2 positive	6 (15)	19 (15)

### Discriminatory SNPs

Germline DNA was obtained and SNP genotyping performed to yield 695,277 SNPs. The analysis confirmed the importance of the main discriminatory SNPs (Table 2). Using the filtering sequence, after we determined and rank ordered the most discriminatory SNPs (*n* = 400), we identified the smallest number of SNPs that were most predictive of risk using the LOOCV algorithm described above. This method's overall predictive accuracy was calculated by iterating all of the filtered samples. We found that a signature consisting of 70 specific SNPs had the highest predictive accuracy of 75.93% (Table 2). Analysis of the SNP signature's predictive accuracy (Figure 2) demonstrated a median accuracy and true positive rate of 75.6% with an interquartile range of 7.3% and a true negative rate of 76.2%. The predictive accuracy's mean and standard deviation was 74.6% and 5.8%, respectively. The minimum and maximum accuracy of these random holds was 54% and 100%, which implies that the minimum size signature of SNPs is quite stable. While we cannot analyze how this predictive signature would vary when new patient data is acquired, we hypothesize the result will be similar to that described by the hold‐out experiments.

**Table 2:** The seventy SNPs that defined AIA risk within the study population, and their possible functional relevance

SNP	SNP Consequence	Impacted Gene	Gene's Relevant Phenotype Attributions	Evidence/Comments
rs72765615	Intron Variant, 3′ UTR Variant	CHD2	–	
rs17149310	Downstream Gene Variant	CFAP77	–	
rs8028334	Intron variant	IL16	RA pathophysiology	Differentially elevated in synovial fluid from RA patients 25, 26, 44, 45, 46, 47, 48 and mediates chemoattraction of CD4+ cells to synovial tissue 25, 26, 49, 50. However, IL16 correlation with clinical disease activity has been conflicting^45^, ^46^.
rs12004732	Intron variant	PLAA	RA pathophysiology	Detected in high levels in RA synovial fluid 22 and may have inflammatory roles 51. Intrathecal injection in rabbit joints results in inflammatory arthritis^21^
rs2883917	Intron Variant	NR3C2	‐ Pain sensitization‐ Fibromyalgia	May be promote visceral hypersensitivity^36^, and be implicated in pathophysiology of fibromyalgia^37^
rs61363926	Noncoding Transcript Exon Variant	BANF1P2	–	
rs56335940	Intron Variant	LINC00882	–	
rs3749817	Missense	FSTL4	–	
rs879605	Intron variant, upstream variant 2KB	LTBR	RA pathophysiology	Induces RA synovial fibroblast proliferation and expression of inflammatory elements 28, 29. Associated with pain and disability in RA patients 27
rs879605	Intron variant, upstream variant 2KB	SCNN1A	–	
rs986324	Intron variant	PTCHD1‐AS	–	
rs7017819	Intron variant, Noncoding Transcript Exon Variant	RP1L1	–	
rs12799692	Intron variant	OPCML	–	OPCML may bind opioids 52. It is also a tumor suppressor in and may be a marker of several types of tumors^53^, ^54^
rs4394668	Intron variant	DHRS3	–	
rs10996945	Intron variant	CTNNA3	–	
rs10996945	Intron variant	LOC105378340	–	
rs705226	Intron variant	LOC105374060	–	
rs73042968	Intron Variant	FBLN2	Breast cancer pathophysiology	Loss of FBLN2 expression is associated with breast cancer progression 55
rs1546734	Intron Variant	LOC105377150	–	
rs17270243	Intron Variant	RORA‐AS1	–	
rs17270243	Intron Variant	RORA*	‐ Breast cancer pathophysiology‐ Estrogen metabolism	‐ May suppress breast tumor invasion by inducing SEMA3F^56^.‐ [Conflicting] Is a transcriptional regulator of aromatase 57. Activates aromatase expression in breast cancer cells, likely contributing to proliferation 58
rs5760686	Intron Variant	SGSM1	–	
rs9907168	Intron Variant	CDC42EP4	–	
rs76098632	Intron Variant	FBXL17	Breast cancer marker	May be a potential biomarker for breast cancer therapy^59^
rs2243511	Intron Variant	TMEM50B		
rs2243511	Intron Variant	IFNGR2	RA pathophysiology	‐Significant differences in blood mononuclear cell expression of IFNGR2 was seen in African American RA patients with erosion and those with no erosion 30
rs1012629	Intron Variant	PTPRK	‐ RA pathophysiology‐ Breast cancer pathophysiology	‐Knocking down its encoded receptor impairs migration and invasiveness of RA fibroblast‐like synoviocytes (FLS), which otherwise progress to destroy cartilage and bone. This receptor mediates inflammatory signaling of TGF‐Beta in RA FLS 60.‐ Is a negative regulator of adhesion and invasion of breast cancer cells; its downregulation is associated with worse prognosis 61
rs322960	Intron Variant	TRPV3	Pain sensitization	‐TRPV3 activation senses peripheral pain 24. It accumulates in peripheral nerves and dorsal root ganglion after injury 23.
rs3743160	Intron Variant, 5′ UTR Variant	SLC28A1	Breast cancer pathophysiology	SLC28A1 expression may be implicated breast cancer cell responsiveness to chemotherapy 62, 63
rs797818	Intron Variant	SEMA3A	‐ RA pathophysiology‐ OA pathophysiology	‐Reduced SEMA3A expression in human synovial tissues was associated with RA disease activity 31‐ SEMA3A expression is elevated in osteoarthritic cartilage, and inhibits VEGF's effects 64
rs11670284	Intron Variant	NLRP13	–	
rs2808787	Intron Variant	COL27A1		*Questionable relevance:‐ The temporal association and location of COL27A1 encoded collagen during calcification/transition of cartilage to bone suggests that the collagen is involved in the process. However, no specific roles have been elucidated 65.‐ A SNP in the region of COL27A1 (rs946053) occurred significantly more in a sample of patients with Achilles Tendinopathy, than in the control group 66.
rs2215016	Intron Variant	RGS6	–	
rs3766160 and rs3820071	Missense Variant	CELA2B	Poorly studied.	
rs10511813	Upstream variant 2KB	LOC105376002	–	
rs12127403	Upstream Gene Variant	VHLL	Poorly studied.	
rs10908495	Missense, Noncoding Transcript Variant	GLMP	–	
rs11683506	Intron Variant	SMARCAL1	–	
rs6081792	Intron Variant, Upstream Gene Variant	RIN2		*Questionable relevance:‐Deficiency of Encoded protein causes a congenital syndrome that includes severe joint hyperlaxity and scoliosis^67^.
rs1047312	3′ UTR Variant	SULT1C2	–	
rs17011869	Intron Variant	CNTNAP5	–	
rs818399	Intron Variant	LINC00922	–	
rs11586047	Intron Variant	LOC105371436	–	
rs28964	Intron Variant	SPACA3	–	
rs10900269 and rs11239786	rs10900269 ‐ Intron Variant rs11239786 ‐ Noncoding Transcript Variant, Synonymous codon	BMS1	–	
rs11600377	Noncoding Transcript Exon Variant	MRGPRF‐AS1	–	
rs11600377	Upstream Gene Variant	MRGPRF		*Questionable relevance:‐ MRGPRF is a part of a family of proteins expressed in pain sensory neurons, and may be specifically activated by neuropeptides. However, MRGPRF, specifically, was not found in dorsal root ganglia in this study 68.
rs62525208	Intron Variant	C8orf37‐AS1	–	
rs61782448	Intron Variant	PLEKHM2	–	
rs77413365	Intron Variant	GRIA1	Inflammatory pain sensitization	‐ Trafficking of this Glutamate receptor in the central nervous system plays a role in inflammatory pain (TNF or IFN‐gamma mediated) and neuronal sensitization 34.‐ May also be implicated in inflammatory central sensitization of dorsal horn^33^.‐Intrathecal injection of a painful inflammatory agent altered subcellular distribution of GRIA1, and decreased GRIA1 concentration in dorsal horn 32.
rs1280408	Intron Variant	CGNL1		*Questionable relevance:‐The promoter of this gene may associate with the aromatase gene (due to a heterozygous inversion in chromosome 15q21.2‐3) to cause pathological aromatase and estrogen excess 69, 70.
rs13013882	Splice Region Variant, Synonymous Codon	MROH2A	–	
rs17599018	Intron Variant	GPM6A	Pain sensitization	‐In cortical cultures of neurons, coexpression of GPM6A markedly increased the endocytosis of Mu‐type Opioid Receptors from plasma membrane to intracellular vesicles 35.
rs2269767	Intron Variant	UBFD1	Inflammatory antagonist	‐UBFD1‐encoded protein was identified as a polyubiquitin binder and may regulate NFkB activity through competitive antagonism of the NFkB pathway 38.
rs7024415	Downstream Gene Variant	ENSG00000253400	–	
rs1546734	Intron Variant	ENSG00000242120	–	
rs4785496	Upstream Gene Variant	ENSG00000260605	–	

Seventy SNPs were identified which were associated with AIA risk. In addition to listing those SNPs, the consequence of each SNP is defined as is the gene most impacted by the mutation. Using literature mining as described in the Methods section, we determined the potential implication of genes relative to the development of AIA.

**Figure 3:**
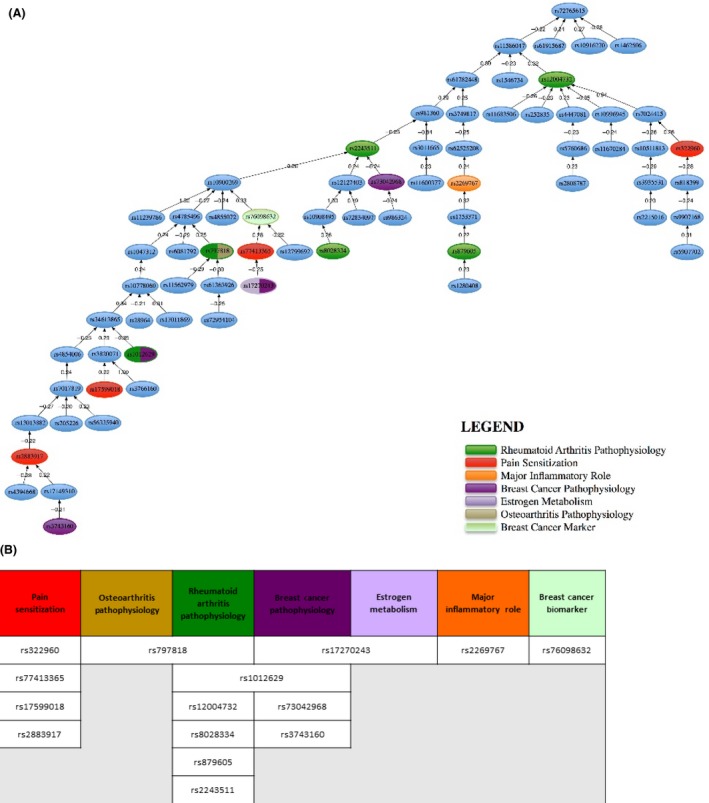
Color visualization delineating the relationship of the 70 predictive SNPs and their functional relevance. (A) Correlation tree of the most discriminatory SNPs. This tree is built using the minimum spanning tree algorithm using the Pearson correlation coefficient. The algorithm looks for the maximum absolute values of the Pearson correlation coefficient (positive and negative correlations) within the set of most discriminatory SNPs. This hierarchical figure describes the strength of relationships between SNPs and how each SNP relates to the others in the cluster.(B) Associated phenotypes include similar phenotypes (RA, pain, inflammation and those associated with the tumor diagnosis).

The four most discriminating SNPs according to their sampling frequency as established by the random sampler were rs1462506, rs17149310, rs2883917, and rs10778060. The correlation network in Figure 3 shows that the header SNP in the graph (rs7276615) is weakly, negatively correlated with rs11586047, rs6195687, rs10916270, and rs1462506. The main tree is developed under rs11586047, and the correlation coefficients are very low, suggesting that these SNPs are almost independent prognostic factors of the arthralgia phenotype. SNPS rs6195687, rs10916270, and rs1462506 are terminal nodes.

### Functional validity and gene attribution

To assess the potential functional validity of the 70 most predictive SNPs, we assessed gene attribution for each SNP and found that 57 genes were associated with the 70 SNPs of interest, with four genes linked to consequences from two or more SNPs (rs12004732 and rs7863476, variants of *PLAA*; rs322960 and rs60292929, variants of *TRPV3*; rs3766160 and rs3820071, variants of *CELA2B*, and rs10900269 and rs11239786, variants of *BMS1*). Assuming that an increase in the ratio of SNP to gene could suggest a more influential impact of arthalgia risk prediction by that gene, we evaluated the relationship between the aforementioned genes and arthalgia‐related phenotypes.

Following the search strategy defined above, we noted that both *PLAA* and *TRPV3* had relevant associations with arthalgia phenotypes. *PLAA* is implicated in inflammatory pathways of synovial cells from Rheumatoid Arthritis (RA) patients 21, 22, and caused inflammatory arthritis when injected into rabbit knee joints 21. *TRPV3* accumulates in peripheral nerves and dorsal root ganglion 23, and its activation is implicated in sensing peripheral pain 24. Associations of *CELA2B* are poorly understood. Lastly, there is no primary data to suggest a direct relationship between *BMS1*, arthralgias or BC, although *BMS1* is also poorly studied. The potential synergistic impact of these genes on AIA risk requires additional study.

In addition to the four genes above, 19 of the other 57 genes associated with the top 70 SNPs were linked to phenotypes involving BC, estrogen metabolism, and significantly, arthralgia. Some of these arthralgia associations include *IL16* (was significantly elevated in synovial fluid of RA patients and mediated chemoattraction of CD4 +  cells into synovial tissues 25, 26), *LTBR* (its expression in RA patients’ synovium positively associated with pain and disability 27, and may be implicated in RA synovial fibroblast proliferation and expression of inflammatory elements 28, 29), *IFNGR2* (joint erosion, joint space narrowing, and disease progression was significantly associated with differential expression of *IFNGR2* in blood mononuclear cells of African American RA patients with radiographic erosion compared to patients with no erosion 30), *SEMA3A* (expressed in synovial tissues and associated with RA 31), *GRIA1* (implicated in inflammatory pain and central sensitization of dorsal horns 32, 33, 34), *GPM6A* (associated with endocytosis of Mu‐type opioid receptors in neuronal cortical cell cultures 35), *NR3C2* (may promote visceral hypersensitivity 36 and may be implicated in pathophysiology of fibromyalgia 37), and lastly, *UBFD1* (implicated as a regulator of NFkB pathway 38) (Table 2).

### Linkage disequilibrium

The LD analysis found 3 sets of SNPs among the top 70 SNP to be in LD. The first LD pair included rs3766160 and rs3820071, missense mutations of *CELA2B*, a poorly characterized gene. The second pair includes LD between rs12127403 (Upstream Gene Variant of *VHLL*, which competetively prevents degradation of HIF‐alpha 39) and rs10908495 (a variant of *GLMP*, another poorly characterized gene). The last pair consisted of rs3011665 and rs981360, which are not known to consequence any specific genes.

## Discussion

AIA is a prevalent and disrupting toxicity of AIs, impacting quality of life, adherence and clinical outcomes 40. As with other regimen‐related toxicities, the risk for AIA is not consistent among patients receiving identical treatments for the same disease. The ability to prospectively differentiate patients at risk for AIA would be desirable at a number of levels, but most importantly in providing actionable data that could inform clinician and patient decision‐making, as well as leading to the design of intervention trials focused on high‐risk individuals. Additionally, the identification of a predictive biomarker strongly associated with a clinically meaningful manifestation of AIA could provide a surrogate for its more accurate reporting.

To identify the SNPs of interest, we used a machine‐learning algorithm for which the SNPs of interest were not pre‐determined. Rather, employing a previously validated technique, we used a filtering step to create a hierarchal list of SNPs most associated with the AIA phenotype while simultaneously eliminating SNPs that were simply genomic “noise” 13. We were able to reduce nearly 400,000 SNPs by three logs to approximately 450 SNPs. We then asked which SNPs were most predictive as a ‘team’ and, using a method in which we sequentially tested every combination of SNPs, identified 70 SNPs that collectively predicted AIA with fair accuracy (75.93%).

Attempts to determine AIA risk‐based strictly on demographic features have been only marginally successful. We found no differences in the study cohorts’ clinical or demographic characteristics. The application of genomic markers, particularly SNPs, as a means to assess toxicity risk associated with cancer treatment regimens has been studied broadly, but with wide ranging and often inconsistent results. In general, two approaches have been used: candidate gene or SNP studies and GWAS. Investigations of genomic risk factors for AIA have exclusively depended on candidate gene/SNP identification.

Since *CYP19A1* codes for the aromatase enzyme in postmenopausal women, it has been an obvious candidate gene for AIA risk prediction 41. While three studies have studied polymorphisms associated with *CYP19A1* relative to AIA and found an association, there is variability in the SNPs reported 12, 41, 42, 43. Other polymorphisms associated with estrogen and vitamin D metabolism have also been targeted. While analyzing such SNPs for risk prediction, Garcia‐Giralt and her colleagues introduced multiplicative terms into their analysis, thus providing a conceptual basis for synergism in contributing to AIA risk 12. In a related investigation, Lintermans et al. reported that a SNP associated with the osteoprotegrin gene was associated with adverse symptoms (hot flashes and pain) in patients treated with AIs 10.

A drawback of candidate gene studies is that they limit discovery of phenotype‐associated genes or SNPs that may not be obvious to investigators deciding on targets. Analogous to trying to describe a landscape in the dark by shining a flashlight with a narrow beam, candidate gene studies may miss important features. Consequently, we took an analytical approach that differed from conventional paradigms in three important ways: (1) we did not mandate a threshold gene expression (SNP) level change for inclusion; (2) we evaluated simultaneous expression of SNP profiles (clusters); (3) the predictive clusters that evolved were driven by their collective and hierarchical relationships with the study groups rather than dependent on preconceptions of an expected result. Importantly, we were able to confirm the relevance of the discovered clusters by confirming their fit into known ontological pathways.

To determine the functional validity of the SNPs in the cluster, we attributed SNPs to their related genes (*n* = 57) and evaluated relevance to several phenotypes that we thought may be expressed in the study cohort. Many individual SNPs were functionally specific for arthralgia‐like disease phenotypes. Others were associated with estrogen metabolism – a finding that supports the hypotheses suggested by the *CYP21A* candidate studies. None of the SNPs in this cluster were attributable to *CYP21A* genes, which may be a component of faults in SNP and gene designation. Genes associated with pain sensitization were notable, a finding theoretically similar to the interests of Lintemans et al 10. Interestingly, genes associated with RA pathophysiology were relatively high in the cluster, suggesting a common biological pathway with AIA and in congruence with other findings implicating an inflammatory mechanism. One SNP in the cluster was closely aligned with inflammation.

Although we included internal cross‐validation in this study, the investigation was limited by small sample size and our inability to have an independent validation cohort. We believe that increasing the training sample will result in a more robust predictive accuracy. An independent validation cohort will be critical to understanding the true clinical meaningfulness and translatability of our findings. A prospective study is currently underway to address these shortcomings.

Nonetheless, we believe that this trial demonstrates the potential utility of an undirected, machine‐learning approach in the development of a predictive test for AIA risk. Such a personalized model in which at‐risk patients are identified prior to therapy start may help to minimize toxicity by prompting the early institution of preventative, therapeutic, or alternative interventions, and thus improve treatment adherence and disease outcomes.

## Conflict of Interest

Disclosures outside the submitted work are noted. Stephen Sonis, Ohio State University (grants), Primary Endpoint Solutions LLC (equity), Biomodels LLC (employee), Inform Genomics (equity); Juan Luis Fernández‐Martínez Primary Endpoint Solutions LLC (consultant); Ana Cernea Primary Endpoint Solutions LLC (consultant); Enrique J. de Andrés‐Galiana Primary Endpoint Solutions LLC (consultant).
